# Subsecond dopamine fluctuations do not specify the vigor of ongoing actions

**DOI:** 10.1038/s41593-025-02102-1

**Published:** 2025-11-10

**Authors:** Haixin Liu, Riccardo Melani, Marta Maltese, James Taniguchi, Akhila Sankaramanchi, Ruoheng Zeng, Jenna R. Martin, Nicolas X. Tritsch

**Affiliations:** 1 Neuroscience Institute, New York University Grossman School of Medicine and Fresco Institute for Parkinson’s and Movement Disorders, New York University Langone Health, New York, NY USA; 2 Department of Psychiatry, Douglas Hospital Research Centre, McGill University, Montreal, Quebec Canada; 3 Integrated Program in Neuroscience, McGill University, Montreal, Quebec Canada

**Keywords:** Basal ganglia, Neural circuits

## Abstract

Dopamine (DA) is essential for the production of vigorous actions, but how DA modifies the gain of motor commands remains unclear. Here we show that subsecond DA transients in the striatum of mice are neither required nor sufficient for specifying the vigor of ongoing forelimb movements. Our findings have important implications for our understanding of how DA contributes to motor control under physiological conditions and in Parkinson’s disease.

## Abstract (web summary)

Liu and colleagues show that the vigor (that is, speed and amplitude) of dexterous movements is not controlled by ongoing fluctuations in extracellular dopamine within the dorsal striatum of mice.

## Main

**Extended Data Fig. 1:**
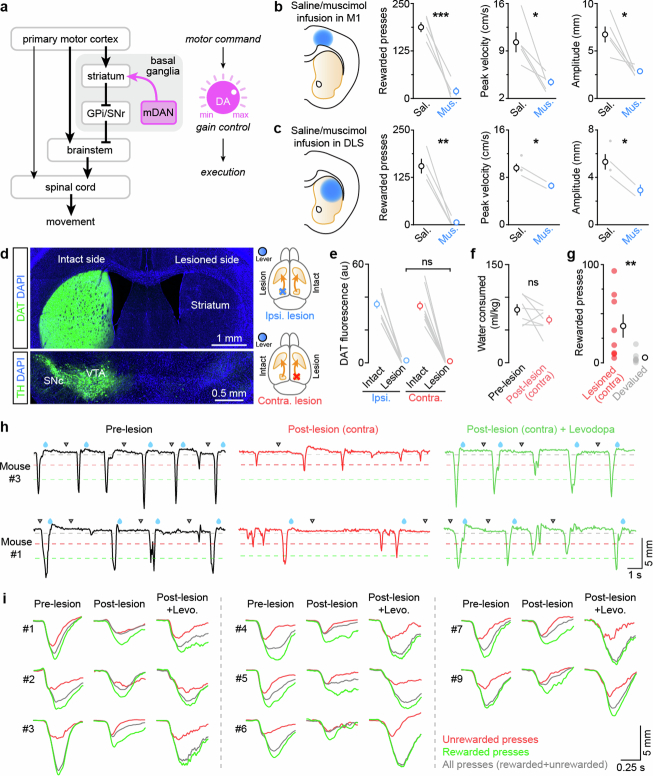
Lever press performance depends on contralateral cortico-striatal circuits. **a**, Model under investigation, in which DA control the moment-to-moment vigor of actions specified in motor cortex. In this model, cortical motor commands are conveyed to the basal ganglia, where fluctuations in striatal DA dynamically modify the excitability of striatal neurons and/or the strength synapses, effectively modifying the gain of cortico-striatal transmission on subsecond timescales. Striatal activity is relayed to the brainstem via the globus pallidus pars interna (GPi) and substantia nigra pars reticulata (SNr). Brainstem circuits integrate motor command and vigor signals to produce vigorous actions via spinal control of the musculature. **b**, *Left*, experimental design: saline or muscimol were infused on separate days in the primary motor cortex (M1) in the right hemisphere (contralateral to the lever-pressing forelimb). *Right*, task performance upon saline (Sal.) or muscimol (Mus.) infusion in M1 (N = 5 mice; number of rewarded presses: p = 0.0009; peak velocity: p = 0.0181; maximum amplitude: p = 0.0159; all paired t-tests). Gray lines: individual mice. Population mean ± SEM shown in black and blue. **c**, Same as **b**, for infusions in dorsolateral striatum (DLS; N = 4 mice; number of rewarded presses: p = 0.0047; peak velocity: p = 0.0410; maximum amplitude: p = 0.0470; all paired t-tests). Note that 2 out of 4 mice failed to produce sufficient presses for quantification in muscimol. **d**, *Left*, fluorescence images of a 6OHDA-lesioned mouse forebrain (*top*) and ventral midbrain (*bottom*) in the coronal plane immunostained for DAT and TH to label DA axons and cell bodies, respectively. SNc, substantia nigra pars compacta; VTA, ventral tegmental area. *Right*, location of 6OHDA infusions relative to the lever-pressing forelimb. **e**, DAT immunofluorescence in intact vs. lesioned dorsal striatum of mice with 6OHDA lesions ipsilateral (blue; N = 7) or contralateral (red; N = 8) to the left forelimb. Note the similar extent of lesions across cohorts (p = 0.12; unpaired t-test). Gray lines: individual mice. Population mean ± SEM shown in black and red. ns: not significant. **f**, Amount of free water consumed by restricted mice before (black) and after (red) lesioning mDANs in the right hemisphere (N = 7 mice; p = 0.16, paired t-test). Population mean ± SEM shown in black and red. Gray lines, individual mice; ns: not significant. **g**, Number or successful presses performed per behavioral session by water-restricted mice with mDANs lesioned in the right hemisphere (red; N = 8 mice) vs. mice for which water was devalued by allowing free access prior to the behavioral session (gray; N = 7 mice; p = 0.0056, Mann-Whitney). Population mean ± SEM shown in black. **h**, Example lever traces from 2 mice before (black) and after (red) lesioning mDANs, and following acute levodopa treatment (green). Deflections in the negative direction indicate inward presses (that is, towards the midline). Triangles depict trial start and blue drop reward delivery. Detection, press and reward thresholds are shown as gray, red and green dashed lines, respectively. **i**, Average lever press trajectories for all presses (that is larger than press threshold; gray), all non-rewarded presses (red) and all rewarded presses (green) across all mice (N = 8) used in contralateral lesion experiments. Source data

Ever since the discovery that degeneration of midbrain dopamine (DA) neurons (mDANs) projecting to the striatum underlies bradykinesia in Parkinson’s disease (PD), DA has become synonymous with motor vigor^1^. However, the mechanisms through which DA contributes to the vigor (that is, speed and amplitude) of voluntary movements are still debated^2,3^. Initial investigations suggested a permissive role, since few mDANs modulate their firing during skilled movements^4,5^, and DA receptor agonists alleviate motor impairments in PD, albeit incompletely^6,7^. Still, the effects of DA on movement may be graded, as prolonged supra-physiological stimulation of DA receptors evokes excessive movements^8,9^. Recent experiments revealed that the activity of mDANs increases at locomotion onset and reflects kinematic parameters^10–16^. These phasic, subsecond changes in striatal DA were proposed to control the gain of motor commands issued by motor cortex, which are relayed to brainstem and spinal circuits via the basal ganglia (Extended Data Fig. 1a). Indeed, DA can modify the strength of cortico–striatal synapses and the excitability of striatal projection neurons^17^. However, the time course and magnitude of such modulatory actions are poorly defined in vivo^18^. For instance, it remains unclear whether subsecond fluctuations in DA can modulate synaptic transmission with sufficient speed to adjust the gain of motor commands on a moment-to-moment basis.

**Fig. 1:**
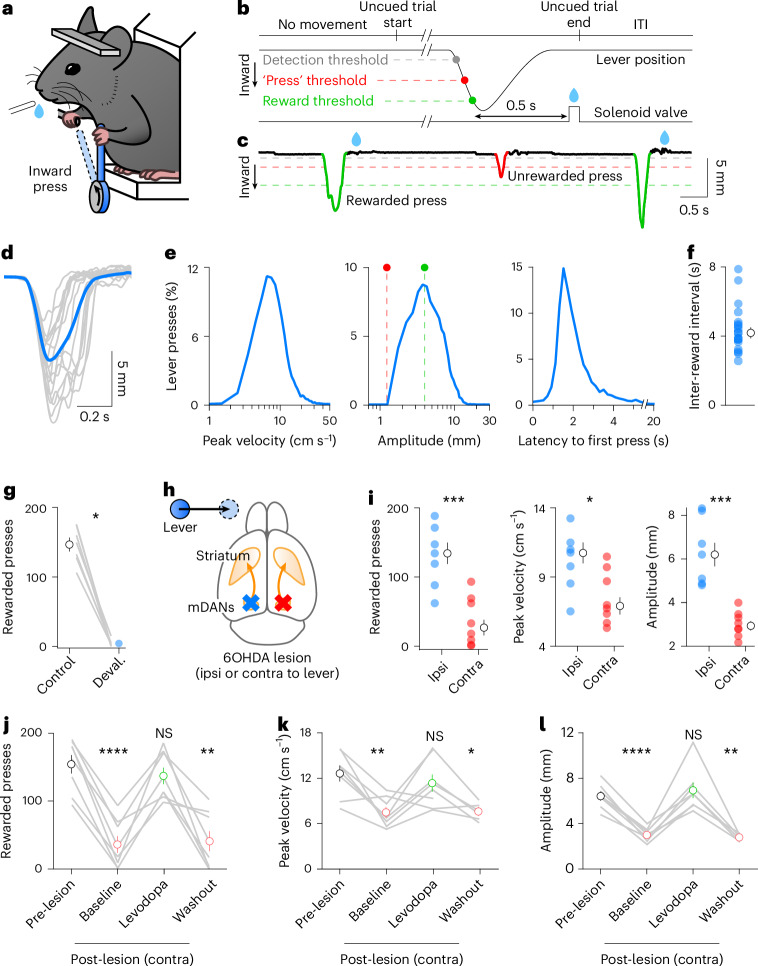
Vigorous execution of self-paced movements requires contralateral DA. **a**, Experimental setup. **b**, Trial structure: mice hold the lever above the detection threshold (gray) for at least 300 ms before pressing the lever inward using their left forelimb. Deflections large enough to cross the reward threshold (green) within 0.5 s trigger delayed delivery of water rewards. To be considered a press, lever deflections must exceed the ‘press’ threshold (red). **c**, Example lever trace. Blue drops: rewards. **d**, Example presses from one mouse aligned to detection threshold crossing (gray: individual presses; blue: session mean). **e**, Probability distribution of peak velocity (left), maximum amplitude (middle) and latency to first press from last reward delivery (right) for all recorded presses (40 sessions in 18 mice). Dashed lines: ‘press’ (red) and ‘reward’ (green) thresholds. **f**, Mean inter-reward interval across 18 mice (blue) and population (black). **g**, Mean number of rewarded presses at baseline (black) and following devaluation (blue; *N* = 7, *P* = 0.0156, Wilcoxon test). **h**, Top-down schematic of mouse brain showing 6OHDA injection sites in separate cohorts to produce mDAN hemilesions ipsilateral (blue) or contralateral (red) to lever-pressing forelimb. **i**, Left: number of rewarded presses from mice lesioned ipsilaterally (blue; *N* = 7) or contralaterally (red; *N* = 8; *P* = 0.0009, unpaired *t*-test). Same for median peak velocity (middle; *P* = 0.0274, unpaired *t*-test) and press amplitude (right; *P* = 0.0008, unpaired *t*-test). **j**, Number of rewarded presses before (black) and after lesioning mDANs contralateral to the lever-pressing forelimb in the absence (red) or presence (green) of levodopa (*N* = 8; treatment: *P* < 0.0001, one-way ANOVA; post-lesion baseline, *P* < 0.0001; post-lesion levodopa, *P* = 0.19; post-lesion washout, *P* = 0.0017, all post hoc Dunnett’s tests versus pre-lesion). **k**, Same as in **j** for median peak press velocity. Note that two mice in the washout group produced too few presses for analysis (treatment: *P* = 0.0024, mixed-effect model; post-lesion baseline, *P* = 0.0069; post-lesion levodopa, *P* = 0.62; post-lesion washout, *P* = 0.049, all post hoc Dunnett’s tests versus pre-lesion). **l**, Same as in **k** for median press amplitude (treatment: *P* < 0.0001, mixed-effect model; post-lesion baseline, *P* < 0.0001; post-lesion levodopa, *P* = 0.84; post-lesion washout, *P* = 0.0021, all post hoc Dunnett’s tests versus pre-lesion). Gray lines in **g** and **j–k** reflect paired data per mouse. Group summary data are mean ± s.e.m. ITI, inter-trial interval; NS, not significant. Source data

To evaluate the role of striatal DA in the expression of motor vigor, we trained head-restrained mice (*N* = 18) to perform self-paced presses of a loaded lever with their left forelimb (Fig. 1a), using a task and manipulandum inspired by others^19–22^. Presses in the inward direction exceeding a minimum amplitude (mean ± s.e.m.: 4.1 ± 0.2 mm) were rewarded with delayed delivery (0.5 s) of a water reward (Fig. 1b,c). Well-trained mice produced ballistic presses every few seconds, 64 ± 5% of which were rewarded (Fig. 1d–f). Importantly, mice stopped producing large-amplitude presses if rewards were devalued by giving mice free access to water before the experiment (Fig. 1g), indicating that the behavior is goal-directed.

To verify that this task relies on cortico–striatal circuits, we silenced neural activity in the forelimb region of primary motor cortex (M1; *N* = 5 mice) or dorsolateral striatum (DLS; *N* = 4 mice) in the right hemisphere (that is, contralateral to the lever-pressing forelimb) using local infusion of the GABA_A_ receptor agonist muscimol. In both cases, muscimol impaired the ability of mice to produce rewarded presses compared with saline (Extended Data Fig. 1b,c), confirming the requirement for contralateral cortico–striatal circuits.

We next assessed the dependence of this task on striatal DA. Early in PD, bradykinesia manifests contralaterally to the hemisphere with the most severe loss of DA^23^. To determine whether our task relies on the integrity of DA axons, we lesioned mDANs unilaterally in either the right or left hemisphere with 6-hydroxydopamine (6OHDA; Fig. 1h and Extended Data Fig. 1d). Despite both groups showing similar denervation (Extended Data Fig. 1e), mice with contralateral lesions (*N* = 8) were significantly impaired in their ability to produce vigorous presses compared with mice with ipsilateral lesions (*N* = 7; Fig. 1i). These data are consistent with a lateralized influence of DA on movement, not a global reduction (a) in the motivation to act, or (b) in the perceived value of rewards. Indeed, mice were similarly motivated to consume water before and after lesioning mDANs (Extended Data Fig. 1f) and produced considerably more presses after lesion compared with after water devaluation (Extended Data Fig. 1g). Moreover, acutely elevating DA levels with levodopa—the first-line therapeutic in the treatment of bradykinesia in PD—temporarily enabled mice with contralateral mDAN lesions to produce large and forceful presses again (Fig. 1j–l and Extended Data Fig. 1h,i). These data suggest a specific role for striatal DA in controlling the vigorous performance of contralateral forelimb movements, and provide our assay with face validity to reveal how mDANs promote motor vigor.

**Fig. 2:**
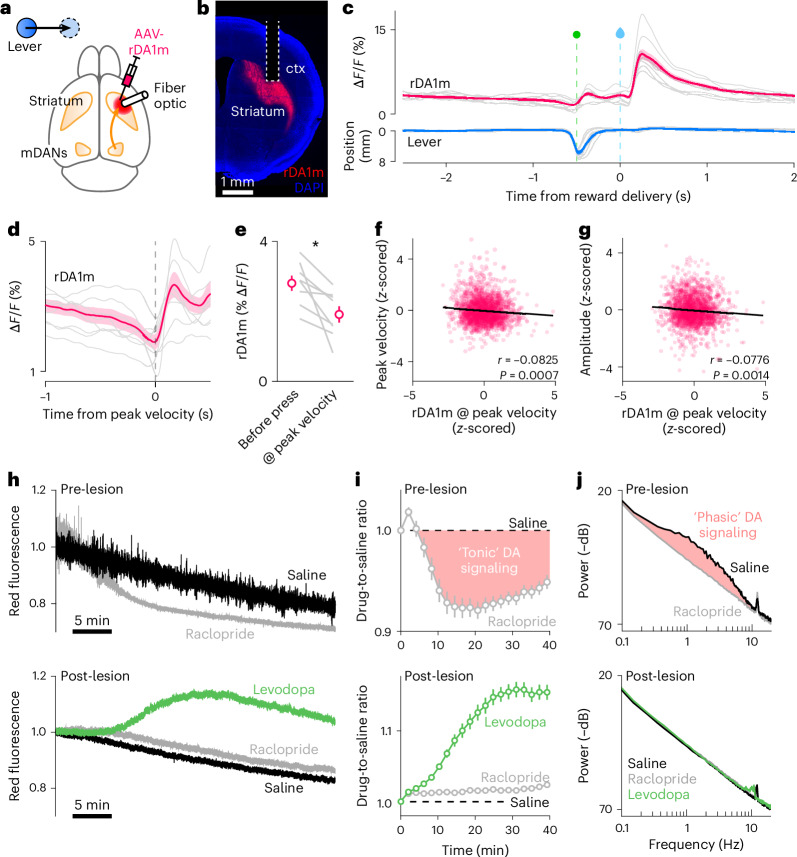
Striatal DA is required for the production of vigorous movements. **a**, Experimental setup. **b**, Coronal brain section showing rDA1m expression and fiber optic track (dashed line) in DLS. Blue: DAPI nuclear stain. All experimental subjects were processed similarly to confirm viral expression and fiber placement in DLS (see summary in Extended Data Fig. 2b). **c**, Mean rDA1m signal (top) and lever trajectory (bottom) aligned to reward delivery (dashed blue line). Data from individual mice shown in gray, population mean (± s.e.m.) in red (top) and blue (bottom; *N* = 8). Dashed green line: time that reward threshold is crossed. **d**, Same as in **c** for rDA1m signal only, aligned to peak press velocity. **e**, Mean rDA1m signal 0.5 s before press onset versus at peak press velocity (*N* = 8; *P* = 0.0134, paired *t*-test). Gray, individual mice; red, population mean ± s.e.m. **f**, Scatter plot of peak press velocity versus instantaneous rDA1m signal at peak press velocity for all recorded presses (*n* = 1,702 across 8 mice). Regression line (black), correlation coefficient *r* and statistical significance of slope being different from 0 (*F*-test) are shown. **g**, Same as in **f** for maximum press amplitude. **h**, Example rDA1m recording (normalized to initial value) in DLS following systemic saline (black), raclopride (gray) or levodopa (green) before (top) and after (bottom) lesioning ipsilateral mDANs. **i**, Ratio of fluorescence signals from **h** (gray, raclopride-to-saline ratio; green, levodopa-to-saline ratio) in 2-min bins before (top) and after (bottom) mDAN lesions. Dashed line indicates ratio of 1. Pink-shaded area highlights fraction of rDA1m signal attributed to ‘tonic’ activation of rDA1m by ambient DA. Data points and error bars are population mean ± s.e.m. (*N* = 8 mice). **j**, Mean power spectrum density of rDA1m signal before (top) and after (bottom) lesioning mDANs (*N* = 8). 1/frequency noise appears as a negatively sloped line. Pink-shaded area reflects low-frequency rDA1m fluctuations, including ‘phasic’ subsecond DA transients typically associated with salient sensory or motor events. Note the absence of subsecond rDA1m oscillations after lesion. Ctx, cortex. Source data

**Extended Data Fig. 2:**
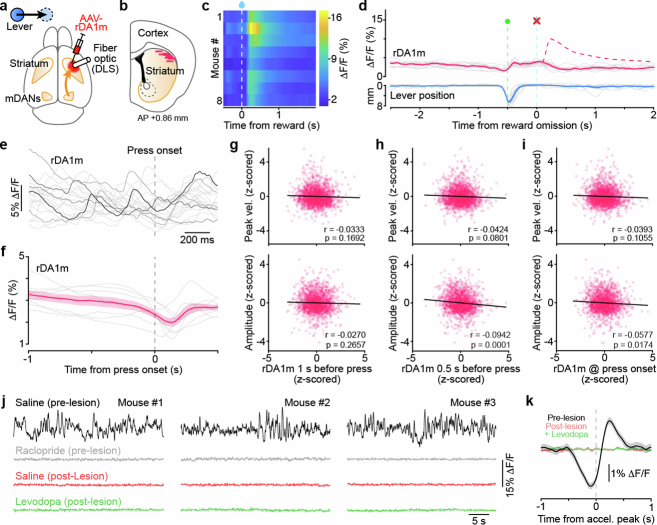
Dorsolateral DA dynamics during lever pressing and in lesioned mice. **a**, Experimental design: extracellular DA levels are monitored in the dorsolateral striatum contralateral to the limb used to press the lever using fiber photometry of the red DA sensor rDA1m virally expressed in striatal neurons. **b**, Fiber optic placement in dorsolateral striatum across N = 8 mice. **c**, Heat plot of average rDA1m fluorescence aligned to water delivery (dashed white line) across N = 8 mice, each showing a brief, short-latency, reward-related increase in fluorescence. **d**, rDA1m fluorescence signal (*top*) and lever trajectory (*bottom*) aligned to reward omission (dashed blue line). Session-average for each mouse (N = 8) shown in gray, and population mean ± SEM shown in red (*top*) and blue (*bottom*). Dashed green line indicates time that reward threshold is crossed. Dashed red line depicts mean rDA1m signal to reward delivery, as shown in Fig. 2c. Note the relative dip in fluorescence at reward omission, and the absence of consistent elevations in DA prior to deflection of the lever. **e**, Example single-trial rDA1m fluorescence signals (each in various shades of gray) aligned to press onset (dashed line). Note the presence of large subsecond fluctuations in DA while mice are immobile prior to press onset, and the absence of consistent elevations in DA before or at press onset. **f**, Average rDA1m signal aligned to press onset (dashed line) for each of N = 8 mice (solid gray traces) and across entire population ( ± SEM; in red). Note the net tendency for DA levels to dip slightly prior to and during press execution. **g**, Scatter plot of peak press velocity (*top*) or press amplitude (*bottom*) vs. rDA1m signal 1 s prior to press onset for all recorded presses (n = 1,702 across 8 mice). Regression line (black), correlation coefficient *r* and statistical significance for slope being different from 0 (F-test). **h**, Same as **g** for rDA1m signal 0.5 s prior to press onset. **i**, Same as **g** for rDA1m signal at press onset. **j**, Example subsecond rDA1m fluorescence signals for 3 mice across different conditions: pre-lesion + saline treatment (black), pre-lesion + raclopride treatment (gray), post-lesion + saline treatment (red) and post-lesion + levodopa treatment (green). **k**, Mean ± SEM rDA1m fluorescence aligned to peak acceleration of locomotion on a treadmill pre-lesion (black), post-lesion (red) and post-lesion with levodopa treatment (green). Note the absence of net movement-aligned changes rDA1m fluorescence post-lesion, consistent with the absence of movement artefacts and the complete loss of phasic DA signaling, including following levodopa treatment. Source data

To investigate whether moment-to-moment DA fluctuations specify the vigor of ongoing movements, we first characterized the relationship between striatal DA and lever pressing in DA-intact mice. To do so, we expressed the red GRAB-DA reporter rDA1m^24^ in the DLS contralateral to the lever-pressing forelimb and monitored changes in extracellular DA using photometry (Fig. 2a,b and Extended Data Fig. 2a,b). In all mice (*N* = 8), water delivery evoked a subsecond increase in DA, consistent with DA’s role in signaling reward (Fig. 2c and Extended Data Fig. 2c,d). We also observed subsecond DA transients outside reward, including during movement and immobility. Surprisingly, presses were not consistently associated with subsecond increases in extracellular DA: on average, DLS DA levels dipped slightly at movement initiation, reaching a minimum as velocity peaked (Fig. 2c–e and Extended Data Fig. 2d–f). In addition, vigor showed either no, or a weak but significant, negative correlation with DA levels 0.5–1 s before press onset, at press onset or when velocity reaches its peak (Fig. 2f,g and Extended Data Fig. 2g–i).

**Extended Data Fig. 3:**
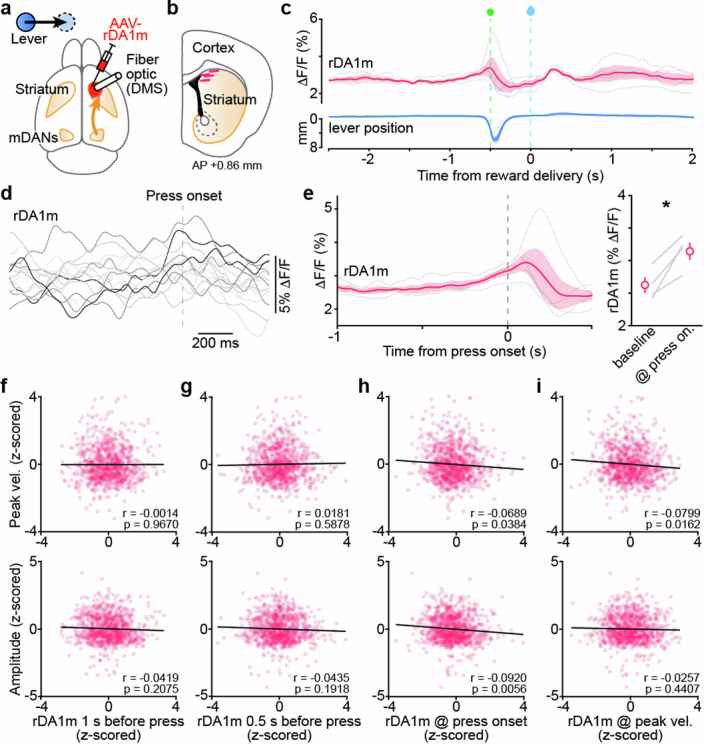
Dorsomedial DA dynamics during lever pressing. **a**, Experimental design: extracellular DA levels are monitored in the DMS contralateral to the limb used to press the lever using fiber photometry of the virally-expressed red DA sensor rDA1m. **b**, Tip of fiber optic placement in DMS (N = 4 mice). **c**, DMS rDA1m fluorescence signal (*top*) and lever trajectory (*bottom*) aligned to reward delivery (dashed blue line). Session-average for each mouse (N = 4) shown in gray, and population mean ± SEM shown in red (*top*) and blue (*bottom*). Dashed green line indicates time that reward threshold is crossed. Note the gradual rise in fluorescence leading up lever pressing and the absence of a pronounced reward response in DMS, both of which are distinct from the kinds of DA transients observed in DLS (see Fig. 2c). **d**, Example single-trial DMS rDA1m fluorescence (each in various shades of gray) from one mouse aligned to press onset (dashed line). **e**, *Left*, session-wide average rDA1m signal in DMS for each of N = 4 mice (solid gray traces) and across the entire population ( ± SEM; in red) aligned to press onset (dashed line). Note the net tendency for DA levels to gradually increase leading up to and following press onset. *Right*, mean rDA1m signal 1 s prior to press onset vs. at press onset (p = 0.0182, paired t-test). Gray: individual mice; red: population mean ± SEM. **f**, Scatter plot of peak press velocity (*top*) and press amplitude (*bottom*) vs. rDA1m signal 1 s prior to press onset for all recorded presses (n = 904 across 4 mice). Regression line (black), correlation coefficient *r* and statistical significance for slope being different from 0 (F-test). **g–i**, Same as **f** for rDA1m signal 0.5 s prior to press onset (**g**), at press onset (**h**) and at peak press velocity (**i**). Source data

To determine how DA levels relate to press vigor in other striatal regions, we repeated rDA1m photometry recordings in another cohort of mice (*N* = 4), this time targeting dorsomedial striatum (DMS; Extended Data Fig. 3a,b). DA dynamics in DMS were qualitatively different from those in DLS around presses: on average, DA levels gradually ramped up leading to press onset and peaked at maximum press velocity (Extended Data Fig. 3c–e). However, similar to DLS, trial-by-trial press vigor did not strongly correlate with DMS DA before press onset, at press onset or when the lever reaches peak velocity (Extended Data Fig. 3f–i). Together, these results suggest that goal-directed forelimb movements are neither reliably preceded by, nor coincident with, subsecond DA transients that scale with motor vigor.

To test whether the production of vigorous actions requires subsecond DA transients, we considered the case of levodopa, whose superior efficacy over other PD therapies is attributed to its ability to restore phasic DA signaling. To assess this, we imaged rDA1m in DLS before and after lesioning mDANs (*N* = 6 mice). In both this experiment and the last (Fig. 1h–l and Extended Data Fig. 1d,e), we lesioned mDANs completely to mitigate concerns that phasic DA release persists in other striatal regions. Pre-lesion, we observed subsecond fluctuations in fluorescence riding on top of a global fluorescence signal (commonly referred to as baseline fluorescence, or *F*_0_) that decreased monotonically over the 40-min-long imaging session, presumably as a result of photobleaching (Fig. 2h and Extended Data Fig. 2j). To estimate what fraction of this baseline signal reflects tonic activation of rDA1m by ambient DA, we systemically administered a submaximal dose of raclopride, a reversible antagonist of D2-type DA receptors, which include the rDA1m sensor. This treatment caused a prolonged decrease in baseline fluorescence relative to saline (Fig. 2h,i), indicating that some of the overall signal stems from steady-state binding of DA to rDA1m (that is, ‘tonic’ DA signaling). Raclopride also reduced the amplitude of subsecond rDA1m transients, confirming that they reflect periodic fluctuations in DA (Fig. 2h,j and Extended Data Fig. 2j). Following complete lesion of mDANs, rDA1m fluorescence no longer showed subsecond fluctuations (Fig. 2h,j and Extended Data Fig. 2j,k) and raclopride no longer depressed baseline fluorescence (Fig. 2h,i), confirming the absence of extracellular DA. Systemic treatment with levodopa evoked a prolonged increase in baseline rDA1m fluorescence (Fig. 2h,i) without subsecond fluctuations (Fig. 2h,j and Extended Data Fig. 2j,k). Together, these data suggest that levodopa’s ability to alleviate bradykinesia does not require subsecond DA transients, and that some steady level of DA signaling is sufficient for the production of vigorous actions.

**Fig. 3:**
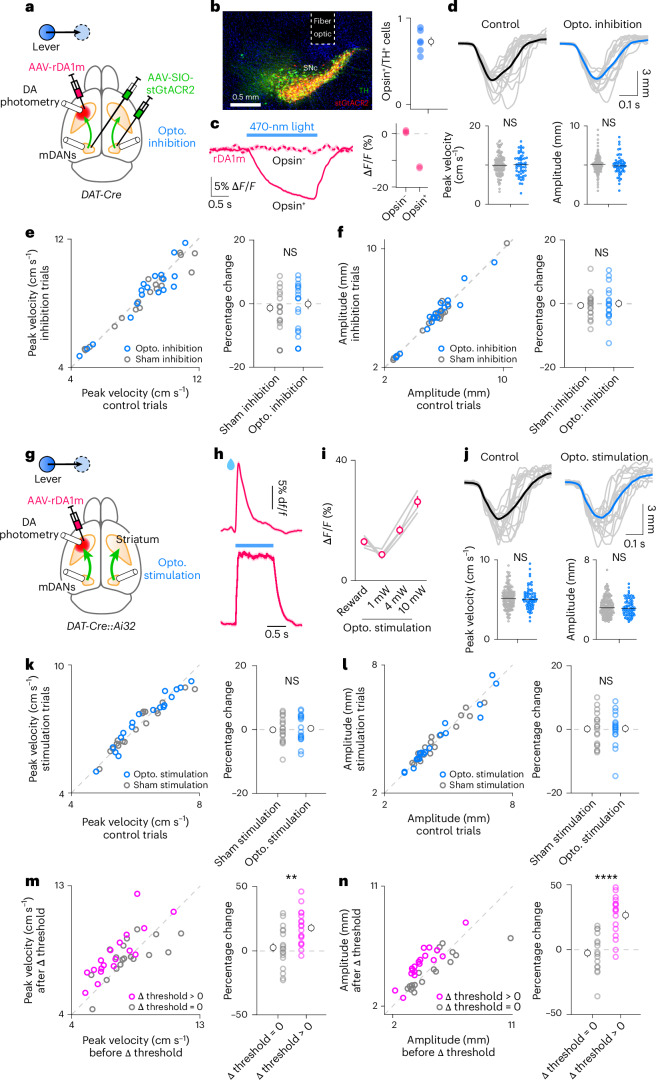
Optogenetic DA manipulations do not affect the vigor of ongoing movements. **a**, Experimental setup for optogenetic inhibition of mDANs. **b**, Left: confocal image of ventral midbrain (coronal plane) showing stGtACR2 (red) in mDANs immunostained for TH (green). Right: mean stGtACR2 penetrance in 11,243 mDANs from *N* = 6 mice. **c**, Left: example rDA1m signal recorded in DLS during blue light delivery (470 nm, 10 mW for 2 s) in the midbrain of mice expressing stGtACR2 (opsin^+^) or not (opsin^−^). Right: summary plot of mean change in rDA1m fluorescence. **d**, Top: representative lever trajectories from one example session (thick line, session-wide mean) for presses occurring on trials with optogenetic light off (left) or not (right). Bottom: peak velocity and amplitude of all presses performed during that example session, separated between control trials (gray; *n* = 127 presses) and mDAN inhibition trials (blue; *n* = 55 presses; velocity: *P* = 0.74; amplitude: *P* = 0.58, two-sample *t*-tests). Black bar, session median. **e**, Left: scatter plot of median peak press velocity for trials with mDAN inhibition (30%) versus trials without (70%) within the same session. Right: same data expressed as a change in peak press velocity for each session. Blue, mDAN inhibition sessions using blue light on 30% of trials (*n* = 18 sessions from 6 mice, 3 sessions each); gray, sham inhibition sessions using no light (*n* = 18 sessions from the same 6 mice, 3 sessions each; *P* = 0.48, mixed-effect model). **f**, Same as in **e** for press amplitude (*P* = 0.70, mixed-effect model). **g**, Experimental setup for optogenetic activation of mDANs. **h**, Example rDA1m signal during reward (top) and optogenetic mDAN activation (bottom; 470 nm, 4 mW, 10-ms pulses at 30 Hz for 1 s). **i**, Mean (± s.e.m.) DA transient amplitude (*N* = 4 mice). Individual mice shown in gray. **j**, Same as in **d** for one example session in which mDANs are optogenetically activated on ~30% of trials (control: *n* = 165 presses; optogenetic stimulation: *n* = 70 presses; peak velocity: *P* = 0.89; amplitude: *P* = 0.56, two-sample *t*-tests). **k**, Same as in **e** for optogenetic activation of mDANs. Blue, stimulation sessions using blue light (*n* = 18 sessions from 6 mice, 3 sessions each); gray, sham sessions using red light (*n* = 18 sessions from the same 6 mice, 3 sessions each; *P* = 0.83, mixed-effect model). **l**, Same as in **k** for press amplitude (*P* = 0.53, mixed-effect model). **m**, Same as in **e** before versus after mid-session change in reward threshold. Magenta, sessions in which threshold is increased (Δ threshold > 0); gray, control sessions in which threshold is kept constant (Δ threshold = 0; *P* = 0.0023, mixed-effect model). **n**, Same as in **m** for press amplitude (*P* = 6.3 × 10^−7^, mixed-effect model). In **b**, **e**, **f** and **k**–**n**, summary data (black) are population mean ± s.e.m. Example traces in **c** and **h** are the mean ± s.e.m. of a minimum of 10 repeats. Opto., optogenetic. Source data

**Extended Data Fig. 4:**
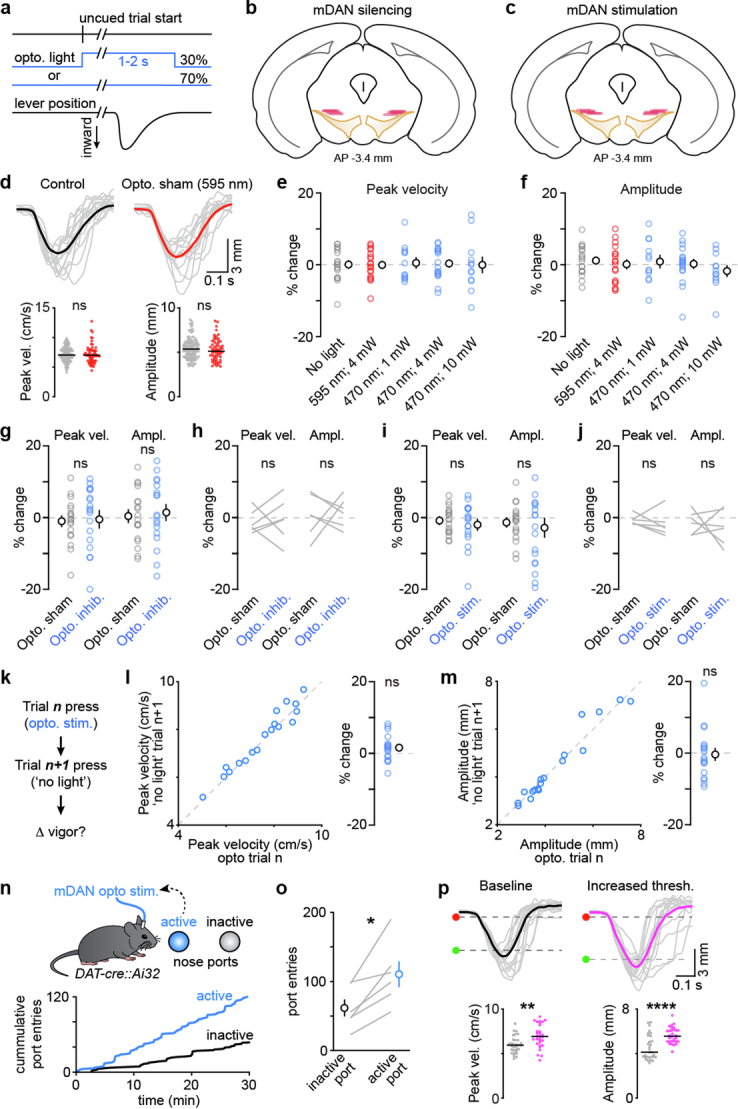
Controls for optogenetic manipulations and sensitivity of behavioral assay to changes in vigor. **a**, Experimental paradigm; DA levels are optogenetically increased or depressed at trial start on 30% of trials using 470 nm light (1 s for optogenetic stimulation, or 2 s for optogenetic inhibition). Lever deflections initiated on those trials are compared either to the 70% of interleaved trials without light (control trials; within-session comparison) or to 30% of trials on separate behavioral sessions without any light delivered to the midbrain or 595 nm light, which does not activate blue light-shifted opsins such as ChR2 and GtACR2 (‘sham’ trials). **b**, Schematic of coronal mouse midbrain section showing bilateral fiber optic placement (red lines) for silencing mDANs (orange). **c**, Same as **b** for mDAN stimulation experiments. **d**, *Top*, Representative lever trajectories from one example session (thick line: session-wide mean) for presses occurring on trials with sham optogenetic light (595 nm) off (*left*) or on (*right*). *Bottom*, peak velocity and amplitude of all presses performed during that example session, separated between control trials (gray; n = 147 presses) and sham stimulation trials (blue; n = 62 presses; velocity: p = 0.90; amplitude: p = 0.32, two-sample t-tests). Black bar: session median. **e**, Within-session change in median peak press velocity between control trials and the ~30% of trials in which optogenetic manipulations are delivered to the midbrain of *Dat-Cre::Ai32* mice: No light (p = 0.96 vs. 0% change, one-sample t-test; n = 18 sessions from 6 mice), 595 nm light (4 mW; p = 0.95 vs. 0% change, one-sample t-test; n = 18 sessions from 6 mice), 470 nm light (1 mW; p = 0.74 vs. 0% change, one-sample t-test; n = 12 sessions from 4 mice), 470 nm light (4 mW; p = 0.73 vs. 0% change, one-sample t-test; n = 18 sessions from 6 mice) and 470 nm light (10 mW; p = 0.98 vs. 0% change, one-sample t-test; n = 12 sessions from 4 mice). Population means ± SEM are shown in black. No difference was observed between conditions (treatment: p = 0.99, one-way ANOVA). **f**, Same as **e** for press amplitude (No light: p = 0.24; 595 nm light: p = 0.90; 470 nm light, 1 mW): p = 0.61; 470 nm light, 4 mW: p = 0.83; 470 nm light, 10 mW: p = 0.26; all vs. 0% change, one-sample t-tests; treatment: p = 0.63, one-way ANOVA). **g**, Re-analysis of data presented in Figs. 3e and 3f using only lever presses initiated during the last 1.5 s of optogenetic light presentation, when striatal DA levels are lowest (n = 18 sessions from 6 mice, 3 sessions each; Peak velocity: p = 0.86; Amplitude: p = 0.72; two-sample t-tests). Note higher variance due to fewer qualifying presses per session. Population means ± SEM are shown in black. ns: not significant. **h**, Same as **g** using mice instead of behavioral sessions as the number of observations (N = 6; peak velocity: p = 0.82; amplitude: p = 0.74; paired t-tests). For each mouse, we averaged the median peak velocity or amplitude across 3 behavioral session. Gray lines are individual mice. ns: not significant. **i**, Same as **g** for data presented in Figs. 3k and 3l using only lever presses produced while optogenetic stimulation is on (n = 18 sessions; Peak velocity: p = 0.51; Amplitude: p = 0.62; two-sample t-tests). **j**, Same as **i** using mice instead of behavioral sessions as the number of observations (N = 6; peak velocity: p = 0.47; amplitude: p = 0.56; paired t-tests). **k**, Graphical description of ‘n + 1’ analysis to test if optogenetic stimulation of mDANs (470 nm, 4 mW) on trial n causes a delayed change in motor vigor on the subsequent (n + 1) trial. **l**, *Left*, scatter plot of median peak press velocity for trials with mDAN stimulation (trial n) vs. the following trial (n + 1) within the same behavioral session. *Right*, same data expressed as a change in peak press velocity for each session (n = 18 sessions in 6 *Dat-Cre::Ai32* mice; p = 0.0805 vs. 0% change, one-sample t-test). Population mean ± SEM shown in black. ns: not significant. **m**, Same as **l** for press amplitude (p = 0.8345 vs. 0% change, one-sample t-test). **n**, *Top*, experimental setup: mDAN stimulation (470 nm, 1 mW, 1 s, unilateral) is triggered when mice nose poke in the active port. *Bottom*, cumulative entries in active and inactive ports during a single behavioral session for one task-naïve mouse expressing ChR2 in mDANs, confirming our ability to drive DA release and promote DA-dependent reinforcement. **o**, Total number of entries in active vs. inactive ports (N = 6 mice; p = 0.01, paired t-test). Population means ± SEM shown in black, individual mice in gray. **p**, *Top*, Representative lever trajectories from one example session (thick line: session-wide mean) for presses occurring before (*left*) and after (*right*) an uncued increase in reward threshold (green dot). *Bottom*, peak velocity and amplitude of 30 presses performed before (gray) and after (magenta) the uncued increase in the press threshold required to earn rewards (velocity: p = 0.0020; amplitude: 4.4e^−5^, two-sample t-tests). Black bar: session median. Source data

These findings do not exclude the possibility that brief changes in striatal DA provide an additional ‘online’ gain to descending motor commands. To investigate this, we asked whether briefly increasing or decreasing DA modifies the vigor of concurrent lever presses. We first examined the effects of mDAN silencing using optogenetics. To do so, we expressed the soma-targeted inhibitory opsin GtACR2^25^ in mDANs and bilaterally implanted fiber optics in the midbrain (*N* = 6 mice; Fig. 3a,b and Extended Data Fig. 4b). We first confirmed in a subset of mice that delivering blue light in the midbrain rapidly and reversibly decreases striatal DA using rDA1m photometry (Fig. 3c). Control experiments in mice lacking an opsin confirmed that blue light does not photoactivate rDA1m in this configuration^26^ (Fig. 3c). Next, we inhibited mDANs for 2 s at uncued trial start on ~30% of trials (Extended Data Fig. 4a). This manipulation did not significantly alter the speed or amplitude of lever presses produced on those trials compared with control trials within the same session, or with ‘sham inhibition’ sessions in which blue light was not delivered (Fig. 3d–f). Similar results were obtained when limiting analyses to presses produced when DA levels are lowest (Extended Data Fig. 4g,h).

To test whether vigor is more sensitive to transient increases in DA, we implanted fiber optics in the midbrain of another cohort of *N* = 6 mice expressing the excitatory opsin ChR2^27^ in mDANs (Fig. 3g), and elevated striatal DA by applying blue light pulses for 1 s at trial start on ~30% of trials (Extended Data Fig. 4a,c). We calibrated stimulation intensity in a subset of mice (*N* = 4) to produce one of three responses (measured with rDA1m; Fig. 3h,i) spanning a range of magnitudes observed endogenously (Extended Data Fig. 2e,j). At all intensities tested, ChR2 stimulation failed to modify the vigor of lever presses compared with control trials within the same session, with sham stimulation trials using red light or with sessions without any optogenetic stimulation (Fig. 3j–l and Extended Data Fig. 4d–f). We also did not observe consistent changes in the vigor of presses produced while optogenetic light was on (Extended Data Fig. 4i,j), or on the following trial (Extended Data Fig. 4k–m). Importantly, stimulation was strong enough to reinforce operant behavior in the same mice (Extended Data Fig. 4n,o). Lastly, we confirmed that our assay is sensitive to changes in vigor by subjecting the same mice to behavioral sessions in which the amplitude of presses needed to earn rewards is suddenly increased mid-session (Extended Data Fig. 4p). Mice responded by increasing the speed and amplitude of presses compared with control sessions in which the reward threshold remained stable (Fig. 3m,n). Together, these results indicate that brief increases and decreases in striatal DA are not sufficient to modify the vigor of ongoing forelimb movements.

In this study, we sought to clarify the role of subsecond DA transients in the moment-to-moment control of motor vigor. We focused on self-initiated forelimb movements, as they are strongly affected in PD and commonly used to assess bradykinesia in humans. Consistent with the lateralization of DA function^15,23^, we found that the production of effortful forelimb movements requires contralateral DA. Our photometry experiments show that levodopa acutely restores motor vigor by globally elevating striatal DA (creating what is commonly referred to as a DA ‘tone’) without reinstating the rapid, subsecond fluctuations that normally ride on top of this signal (that is, ‘phasic’ DA transients). This suggests that levodopa’s therapeutic efficacy does not require phasic DA signaling and, in turn, that the production of vigorous movements does not necessitate that DA receptors be briefly engaged in phase with motor actions. Indeed, DA receptor agonists also alleviate bradykinesia, as does levodopa when vesicular monoamine release is abolished^6,7,28^. In addition, mice in which phasic DA release is reduced are viable and do not show gross motor impairments^29,30^, unlike mice in which DA release is fully prevented^31^.

In DA-intact mice, we failed to observed a strong, systematic relationship between the trial-by-trial magnitude of DA transients and motor vigor in either DMS or DLS. However, averaged data revealed a comparatively much smaller but significant dip in DLS DA at peak velocity that scales with vigor, while in DMS, mean DA levels showed a small upward ramp before movement onset. One caveat of population-level photometry is that it may miss highly localized DA transients that could be important for specific aspects of forelimb movements. Still, our findings echo observations made by others during self-initiated actions^10–16,32–38^, suggesting at minimum that DA does not obey a fixed, striatum-wide relationship with respect to motor vigor. DA may instead follow a multitude of release patterns that vary across mDAN subtypes, striatal subregions and task demands; the notion that every movement is universally accompanied by a global elevation in striatal DA is too simplistic and should be resisted.

Our optogenetic experiments further suggest that the vigor of forelimb movements is unlikely to be controlled by concurrent changes in striatal DA. mDAN stimulation and inhibition both failed to modify vigor, irrespective of whether movements were produced during optogenetic manipulations (Fig. 3e,f,k,l and Extended Data Fig. 4g–j), in the seconds that followed (Extended Data Fig. 4k–m) or cumulatively over the course of a session (Fig. 3e,f,k,l). These negative findings do not reflect a failure to alter DA levels: we calibrated manipulations to match or exceed DA transients observed in vivo, broadened their anatomical reach by delivering optogenetic light to the midbrain, confirmed that stimulation is strong enough to reinforce operant behavior and verified that our assay is sensitive to changes in vigor prompted by changes in reward contingency. In addition, our findings are broadly consistent with others that failed to reveal strong instantaneous effects of mDAN manipulations on the vigor of well-isolated, self-paced movements^37–41^.

Together, our data indicate that subsecond DA transients are neither required for producing vigorous forelimb movements, nor sufficient for specifying their vigor on the timescale of seconds. Our findings expand on recent work showing that phasic DA is dispensable for the production of simple bilateral movements in mice, such as licking and locomoting^29,30^, and that changes in mDAN activity are only weakly correlated with the velocity of concurrent and upcoming actions^12,13,35,37^, leaving much of the trial-by-trial variance in motor vigor unexplained by DA (Fig. 2f,g and Extended Data Figs. 2g–i and 3f–i). This should not be interpreted to suggest that DA cannot modify movement under any circumstance. It is well established that elevating DA increases the frequency with which actions are produced and/or decreases the latency to initiate actions^8,9,12,13^. While these effects are broadly consistent with DA invigorating behavior, they may not reflect changes in the speed or amplitude of individual limb movements per se, but changes in motivation, action selection or action initiation^13,42^. It is also possible that supra-physiological changes in DA (in concentration, duration or spatial extend) promote behavioral effects that are not representative of DA under more physiological conditions^37,38^.

What about subsecond DA transients? Can they play any role in modifying the speed and amplitude of individual movements? Elegant studies^21,37,40,43,44^ suggest that they can, following multiple pairings with actions of a given speed and/or amplitude via reinforcement learning. According to this view, glutamatergic synapses repeatedly activated in phase with subsecond DA transients gradually experience synaptic plasticity, thereby sculpting striatal ensembles required for the selection, preparation and/or execution of movements of any vigor. This mechanism is attractive for several reasons: first, it confers DA transients the ability to modify multiple kinematic parameters. Second, it provides DA with the flexibility to reinforce movements that are either more or less vigorous. Lastly, similar plasticity mechanisms have been proposed to contribute to PD’s motor impairments as well as to levodopa’s long-lasting therapeutic effects^45,46^, providing a plausible mechanism for understanding how phasic DA contributes to motor vigor under physiological and pathological conditions.

## Methods

### Animals

All procedures were performed in accordance with protocols approved by the NYU Grossman School of Medicine Institutional Animal Care and Use Committee (protocol no. IA16-02082). Mice were housed in groups before surgery and singly after surgery under a reversed light–dark 12-hour cycle (dark from 6:00 to 18:00). Food and water were provided ad libitum, except when water-restricted to incite consumption of water rewards. Experiments were carried out using both male and female mice maintained on a C57BL/6J background (Jackson Laboratory; cat. no. 000664). For optogenetic experiments, mDANs were targeted using heterozygous *Dat-Cre* mice (Jackson Laboratory; cat. no. 006660). The inhibitory opsin stGtACR2 was expressed virally while the excitatory opsin ChR2 was expressed genetically by crossing *Dat-Cre* and *Ai32* (Jackson Laboratory; cat. no. 012569) mice to obtain offspring heterozygous for both transgenes.

### Surgeries

Mice (range: 8–39 weeks of age at the time of surgery; mean: 18 ± 2) were anesthetized with isoflurane, placed in a stereotaxic apparatus on a heating pad and administered ketoprofen (10 mg kg^−1^ in saline) subcutaneously. After exposing and cleaning the skull under aseptic conditions, coordinates (given in mm from bregma) were marked on the skull (M1: anterior-posterior (AP) +0.3, medial-lateral (ML) +1.5; DLS: AP 0, ML +2.8; DMS: AP +0.7, ML +1.3; mDANs: AP −3.1, ML +1.4) and were either used to guide craniotomies or covered with Kwik-Cast (WPI) before applying dental cement (C&B Metabond, Parkell) for future injections. A custom titanium headpost was attached to the skull in the horizontal plane posterior to lambda using a three-dimensionally printed (3D-printed), negative air pressure-based holder and dental cement. To allow for drug infusions in DLS, a cannula (P1 Technologies; cat. no. C315GS-5) was implanted at a depth of 2.05 mm from dura. To image DA by photometry, 200 nl of an adeno-associated virus (AAV) encoding the red-shifted GRAB-DA sensor rDA1m (AAV9-hSyn-rDA1m; titer: 6.37 × 10^12^; Vigene Biosciences; diluted 1:3 in sterile saline) was infused in the DLS or DMS at a depth of 2.05 mm below dura using a micro-syringe pump (KD Scientific; Legato 111; rate: 100 nl min^−1^) fitted with a Hamilton syringe (cat. no. 1701 N) connected to a pulled glass injection micropipette (Drummond Wiretroll II; 100-µm tip) via polyethylene tubing filled with mineral oil. A 4-mm-long fiber optic cannula (400-μm core, 0.5 numerical aperture (NA), 1.25-mm ferrule, >80% light transmission efficiency; RWD Life Science) was subsequently implanted 0.2 mm above the injection site, vertically for DMS, or with a −5-degree offset (in ML plane) for DMS. To inhibit mDANs optogenetically, 1 µl of AAV1-hSyn1-SIO-stGtACR2-FusionRed (titer 4.5 × 10^11^; Addgene cat. no. 105677; diluted 1:40 in sterile saline) encoding the soma-targeted inhibitory opsin GtACR2 in a Cre-dependent fashion was injected bilaterally at a depth of 4.2 mm below dura. To deliver light to mDANs in these and *Dat-Cre::Ai32* mice, a 6-mm-long fiber optic cannula (400-μm core, 0.5 NA, 1.25-mm ferrule, >80% light transmission efficiency; RWD Life Science) was implanted at a depth of 4.0 mm from dura. Implants were cemented to the skull using C&B Metabond. Mice were allowed to fully recover in their home cage for at minimum 1 week before initiating behavioral training.

### Self-paced lever-pressing task

The behavioral apparatus consisted of a soundproof chamber, an aluminum headpost holder, a 3D-printed enclosure with foam cushion to hold the mouse, a 3D-printed plastic lever with handle^22^ attached at its mid-point (that is, axis of rotation) to an analog rotary encoder (US Digital; MA3 magnetic shaft encoder) and a water-delivery tube connected to a quiet solenoid valve (Lee Company; cat. no. LHQA0531220H). The lever was set to the left of head-fixed mice so as to be easily manipulated along a single axis (toward left or right) with their left forelimb. A horizontal metal bar was provided on the right to rest their right forepaw. The lever was loaded with a pair of magnets (one attached to the bottom of the lever and another, circular magnet affixed to the floor of the chamber) to help maintain the lever near the same resting position and to impose an increasing load on lever deflections of increasing amplitude. Behavioral task parameters were controlled by an Arduino Due micro-processor running an open-source package (Bpod, v.0.5, Sanworks). The voltage signal provided by the lever’s rotary encoder was handled by another Arduino Due; its signal was smoothed online (10-ms median filter) and threshold crossings were determined online using custom Arduino programs and a MATLAB graphical-user interface. Analog and digital signals (for example, lever position, synchronization signals, time-to-live pulses) were digitized at 10 kHz using a National Instruments data acquisition board (cat. no. PCIe-6353) and breakout terminal block (cat. no. BNC-2090A) and recorded using Wavesurfer (Janelia; v.1.0.6). Deflections of the lever in the rewarded (that is, inward) and nonrewarded (that is, outward) directions were read out as negative and positive changes in voltage, respectively.

To incite mice to learn and perform self-paced ballistic presses of the lever, access to water was restricted to the recording rig. Weight was monitored daily to ensure mice maintained >85% of their original body weight. Mice were introduced to the task gradually; they were first habituated to head-fixation and water collection from the lick spout, before being trained to grab the lever with their left forepaw and depress it inward past thresholds of increasing amplitude to obtain water rewards. To initiate a self-paced trial, mice needed to keep the lever still (that is, under the detection threshold; see below) for a minimum of 300 ms (‘no movement’ period; Fig. 1b). Any deflection of the lever during this window caused a resetting of the clock and postponement of trial start. We define lever deflections as any displacement of the lever past the ‘detection threshold’ (±0.5 mm around the lever’s default rest position), which we deliberately kept small to ensure that mice remain still during the ‘no movement’ period and that they initiate trials from similar lever positions. Trials ended after mice obtained water for performing a successful press or 30 s elapsed, whichever occurred first. To be considered a full-fleshed press, the deflection of the lever had to exceed a ‘press threshold’ set 1.7 mm to the right of the lever’s rest position (Fig. 1b). This threshold was established empirically to distinguish intentional presses of the lever from small deflections occurring when mice occasionally adjust their posture/grip by comparing the amplitude distribution of lever deflections in water-restricted mice working to collect rewards versus water-sated mice merely holding the lever. A successful press was defined as any press that crossed both the detection threshold and ‘reward threshold’ (4.1 ± 0.2 mm relative to lever’s rest position) within less than 0.5 s. The reward threshold was set large enough for lever deflections to be deliberate and effortful, but well-within the range of amplitudes that mice can produce (Fig. 1c–e). Unsuccessful presses comprise lever presses (that is, deflections at least 1.7 mm in amplitude) that failed to cross the reward threshold or to meet the timing requirement for earning a reward. Approximately two-thirds of lever presses met the criteria for reward delivery (that is, successful presses) in well-trained mice. Rewards (~5 μl of water) were delivered 0.5 s after crossing of the reward threshold to separate operant from consummatory actions. The end of each trial was followed by a 1-s inter-trial interval to allow for water consumption, during which time lever deflections were not rewarded. Mice reached expert performance (~200 rewarded trials per 30-min session) after approximately 14 daily training sessions, adopting for the most part a successful strategy of self-initiating ballistic deflections of the lever towards the right every few seconds (Fig. 1c,f and Extended Data Fig. 1h). To probe whether performance is goal-directed in Fig. 1g, we devalued rewards by providing mice with free access to water in their home cage for 1 h before behavioral testing. To compare motivation to consume water in restricted mice before and after mDAN hemi-lesion (Extended Data Fig. 1f), mice were individually placed for 10 min in a cage with a dish containing 10 g of water. We measured the amount of water consumed by weighing both mice and water immediately before and after the 10-min period. To ensure hemi-lesioned mice remain engaged in the task and motivated to press the lever, the reward threshold was lowered on a subset of trials to help mice collect some rewards. To probe whether expert mice can adjust the speed and/or amplitude of lever presses mid-session (Fig. 3m,n), we increased the reward threshold by 10% on trial 80 (uncued).

### Muscimol inactivation

To acutely silence neural activity in M1 or DLS, we slowly infused 200 nl of the GABA_A_ receptor agonist muscimol (Tocris; 2 μM in saline) into the target area using a micro-syringe pump (KD Scientific, Legato 111; 50 nl min^−1^) while mice were briefly and lightly anesthetized with isoflurane in a stereotaxic frame. For M1 inactivation, we made two 100-nl injections at 400 μm and 900 μm below dura using a sharp glass pipette (<50-μm tip) through a small craniotomy made over M1 the day previous. For DLS inactivation, we used an infusion cannula (P1 Technologies; cat. no. C315IS-5) through a chronically implanted guide cannula (see above). Infusion cannulas and glass needles were left in place for 10 min after infusion. Mice were then returned to their home cage, where they emerged from anesthesia within minutes. Behavioral sessions started 10 min later. Control sessions using saline instead of muscimol were performed using the same procedure.

### Unilateral mDAN lesions

Mice were given free access to water for at minimum 2 d ahead of surgery, and were administered desipramine and pargyline (Sigma-Aldrich; 25 mg kg^−1^ and 5 mg kg^−1^, respectively) intraperitoneally 1 h before surgery to increase the selectivity and efficacy of 6OHDA. mDANs were lesioned unilaterally with 6OHDA as before^47^. Briefly, upon exposing the previously labeled substantia nigra pars compacta (SNc) coordinates on the skull, a small craniotomy was performed and 3 μg of 6OHDA (freshly dissolved in 200 nl of sterile saline containing 0.2% ascorbic acid) was slowly infused 4.4–4.0 mm below dura at a rate of 100 nl min^−1^ using a pulled glass micropipette. Following surgery, mice were allowed to recover in their cage for at minimum 1 week and were provided with twice-daily injections of a glucose solution (5% w/v in saline, 0.5 ml each; intraperitoneally) and saline (1 ml each; subcutaneous), and once-daily injections of ketoprofen (10 mg kg^−1^ in saline; subcutaneous). Water restriction resumed only once mice fully recovered from the lesion and maintained a stable weight. Mice remained motivated to collect water rewards from the spout after hemi-lesion (Extended Data Fig. 1f) and capable of grabbing the lever and producing inward presses for reward for the duration of behavioral sessions (Fig. 2h–l and Extended Data Fig. 1g,h).

### Pharmacological treatments

Levodopa (Tocris, 1.5 mg kg^−1^; along with the peripheral DA decarboxylase inhibitor benserazide hydrochloride; Sigma-Aldrich, 12 mg kg^−1^) and raclopride (Sigma-Aldrich, submaximal dose of 1 mg kg^−1^ to avoid cataplexy) were administered intraperitoneally in saline. Behavioral experiments (Fig. 1j–l and Extended Data Fig. 1h) were carried out within 10 min of treatment. Photometric imaging of rDA1m was initiated immediately after injection.

### Fiber photometry

Extracellular DA levels were monitored as before^33,48^ using a custom-made photometry system consisting of a fluorescence mini-cube (Doric; cat. no. FMC5_E1(460-490)_F1(500-540)_E2(555-570)_F2(580-680)_S) connected to: (1) a 565-nm fiber-coupled light-emitting diode (LED) (Thorlabs; cat. no. M565F3) via a 400-µm, 0.48 NA fiber optic patch cord (Doric); (2) a photoreceiver (Newport; cat. no. 2151) via a 600-µm, 0.48 NA fiber optic patch cord (Doric); and (3) our specimen via a 400-µm, 0.48 NA fiber optic patch cord (Doric). Excitation light was calibrated to consistently measure ~30 µW at the tip of patch cords, which were selected for their low baseline autofluorescence, and were further ‘photobleached’ by constant exposure to >17-mW blue light for a minimum of 12 h before each recording. To ensure stable light delivery, photometric imaging commenced only after LEDs were turned on for at least 10 min. Voltage signals from the photoreceiver were digitized with a National Instruments data acquisition board (cat. no. PCIe-6353) and breakout terminal block (cat. no. BNC-2090A) at 2 kHz and recorded with Wavesurfer. To characterize the effects of saline, levodopa and raclopride on striatal DA, we imaged rDA1m in mice head-fixed on a treadmill^33^ for 40 min immediately after pharmacological treatment.

### Optogenetics

To inhibit mDANs, mice implanted bilaterally with fiber optic cannulas above stGtACR2-expressing mDANs were connected to 470-nm fiber-coupled LEDs (Thorlabs; cat. no. M470F3) using patch cords (Thorlabs; cat. no. M98L01). Light power was adjusted to measure 10 mW at the tip of the patch cord using a power meter (Thorlabs; cat. no. PM100D and S120C sensor) before each experimental session. As control, we ran sham inhibition sessions in which the 470-nm LED was kept off, such that no stGtACR2 activation occurred. To stimulate mDANs, blue light (470 nm; 1, 4 or 10 mW at the tip of the patch cord) was delivered in the ventral midbrain of *Dat-Cre::Ai32* mice implanted with fiber optic cannulas in SNc. In addition to control sessions in which no light was delivered to the midbrain, we ran sham stimulation sessions on separate days using a 595-nm fiber-coupled LED (Thorlabs; cat. no. M595F2) to control for non-opsin-mediated effects of light on behavior, as ChR2 is not excited at this wavelength. Optogenetic and sham manipulations were applied for 1–2 s at the start of a subset (~30%) of pseudo-randomly selected trials using Wavesurfer starting on trial 11. The net effect and magnitude of optogenetic manipulations on striatal DA levels (Fig. 3c,h) were measured in a subset of mice co-expressing rDA1m and implanted with a fiber optic cannula in DLS. To exclude the possibility that the rDA1m transients imaged during optogenetic manipulations in Fig. 3c,h reflect rDA1m photoactivation^26^, control experiments were carried out in a separate cohort of wild-type (that is, opsin-negative) mice using the exact same blue light stimulation and rDA1m imaging parameters. To test whether ChR2 stimulation is sufficiently strong to promote behavioral reinforcement in Extended Data Fig. 4n,o, mice were placed for 30 min in a custom-built plastic enclosure fitted on one wall with two nose ports equipped with infrared LED sensors (Digi-key Electronics; cat. no. 365-1769-ND) to detect head entries. Mice were connected to a 470-nm fiber-coupled LED (Thorlabs; cat. no. M470F3) via a rotary joint (Thorlabs; cat. no. RJ1) and patch cord (Thorlabs; cat. no. M98L01) and ChR2 was stimulated unilaterally (4 mW, 10-ms pulses at 30 Hz for 1 s) immediately upon head entry into one of the two ports (referred to as the ‘active’ port; counter-balanced across mice) using Wavesurfer. Port entries were recorded using the same program.

### Data processing and analyses

#### Lever press analyses

To determine the speed and amplitude of lever presses, we first identified presses (both successful and not; as defined above) during self-paced trials. In well-trained mice, individual trials typically comprised one or two well-separated ballistic presses of the lever in the rewarded direction, but occasionally contained multiple sequential deflections riding on one another (that is, without returning to the rest position in between), or instances when mice accidentally let go of the lever mid-press, leading to rapid oscillations of the lever back to its resting position. To identify lever presses and quantify their speed and amplitude, we isolated contiguous periods of time during which the instantaneous velocity of the lever remained greater than 5 mm s^−1^ in the rewarded direction (defined as movement segments) using the voltage traces derived from the rotary encoder (down-sampled to 1 kHz and low-pass filtered to 30 Hz, 6-pole Butterworth). We calculated press amplitude as the furthest distance reached by the lever in the inward direction, and peak velocity as the largest instantaneous velocity recorded during either movement segment. To exclude the rare high-velocity oscillatory artefacts caused by mice letting go of the lever, we restricted analyses to movement segments before the lever suddenly travels back to through its resting position and overshoots it by >2.5 mm. In all figures, we report press amplitude and peak velocity as session-wide median values. To best estimate population medians, especially in conditions with few or imbalanced numbers of qualifying presses between test and control groups, we first bootstrapped amplitude and velocity values 1,000 times. The effects of optogenetic and sham manipulations (delivered on ~30% of trials) on press vigor were expressed as a percentage change from the 70% of within-session trials during which no manipulation was performed (that is, ‘control trials’), calculated as (median press vigor during ‘manipulation trials’ − median press vigor during ‘control trials’) × 100/median press vigor during ‘control trials’. Changes in press amplitude and velocity caused by the uncued increase in reward threshold (Fig. 3m,n) were quantified similarly, using the median amplitude and peak velocity of lever presses during 30 consecutive trials before and 200 s after the threshold shift. In figures, percentage changes in vigor were statistically compared either with the null hypothesis of 0% change, or with percentage change values observed on sham stimulation sessions (run in the same mice, on separate days) in which either no light (Fig. 3e,f and Extended Data Fig. 4e,f) or 595-nm light (Fig. 3k,l) is delivered to the midbrain. To assess delayed changes in press vigor taking place a few seconds after mDAN stimulation (Extended Data Fig. 4k–m), we compared presses with optogenetic stimulation (trial *n*) with those performed on the following trial (trial ‘*n* + 1’) in the absence of DA stimulation.

#### Photometry

Voltage signals from the photoreceiver were down-sampled to 1 kHz and low-pass filtered at 30 Hz (10-pole Butterworth). Across subjects, the overall voltage recorded from the red fluorescence channel at the beginning of any recording ranged between 0.7 and 2.0 V. This voltage reflects multiple components: photoreceiver dark noise (minor), patch-cord autofluorescence, brain/vasculature autofluorescence and rDA1m signal. The latter further consists of two components: the low-level basal fluorescence contributed by rDA1m unbound to DA, including rDA1m in intracellular organelles, and the comparatively brighter fluorescence signal brought about by the binding of DA to rDA1m at the plasma membrane. Given that we used the same equipment and LED power across subjects, we attribute most of the variability in starting voltage to differences in rDA1m expression and/or in DA binding, with a minor component originating from day-to-day variability in light transmission efficiency at the patch cord-to-fiber optic cannula interphase. To estimate what fraction of the photoreceiver voltage reflects binding of rDA1m to extracellular DA, we administered the reversible rDA1m antagonist raclopride. We attribute the negative shift in fluorescence shown in the top plot of Fig. 2h to the loss of rDA1m activation by DA since this negative shift is absent in 6OHDA-lesioned mice. Note, however, that raclopride also alters fluorescence readings independent of DA binding to rDA1m; it causes a rapid increase in red fluorescence (most evident in 6OHDA-lesioned mice and during the first few minutes after injection in DA-intact mice; Fig. 2i), possibly as a result of intrinsic fluorescence or changes in hemodynamics. We opted not to correct for this in figure displays (Fig. 2h,i) and analyses for transparency, noting that we are likely underestimating the fraction of the fluorescence signal reflecting DA binding to rDA1m in pre-lesion mice (in addition to the fact that a submaximal dose of raclopride was used).

To enable comparisons between and within animals on different experimental days, as in Fig. 2h, we normalized photoreceiver readings to the average voltage recorded during the first 15 s of any recording. For transparency, we also did not correct for photobleaching in Fig. 2h, which presumably accounts for the near-linear loss of red fluorescence signal across conditions. For analyses in Fig. 2i, we corrected for photobleaching by averaging voltage signals in 2-min bins for each condition, before dividing each bin by the mean voltage recorded in saline-treated mice in the same time bin. By definition, saline-treated recordings adopt a value of 1 at all time points. To quantify the power spectrum density of photometry data in Fig. 2j, we used the open-source MATLAB package Chronux (http://chronux.org) on raw, unfiltered and uncorrected voltage signals from the photoreceiver and plotted power at frequencies ranging from 0.1 to 11 Hz on semi-log plots. These analyses reveal two distinct types of rDA1m fluorescence signals unfolding on different time scales: fast, subsecond fluctuations in extracellular DA (taking place on the order of 0.5–4 Hz)^33^ riding on top of a slowly fluctuating (tens of seconds or more) rDA1m signal. We refer to the latter as a ‘global’ fluorescence signal because it accounts for a considerable fraction of the voltage recorded at baseline. Fast and slow rDA1m signals presumably reflect what is commonly referred to as ‘phasic’ and ‘tonic’ DA receptor signaling.

For experiments combining photometry and optogenetic manipulations with blue light (Fig. 3h), we removed stimulation-locked light artifacts (±6 ms around each light pulse) and filled data by extrapolation using the inpaint_nans function (MATLAB Central) before filtering.

### Immunohistochemistry

Mice were deeply anesthetized with isoflurane and perfused transcardially with 4% paraformaldehyde (Electron Microscopy Sciences) in 0.1 M sodium phosphate buffer. Brains were post-fixed overnight and sectioned coronally (50–100 μm) using a vibratome (Leica; cat. no. VT1000S). Brain sections were mounted on superfrost slides and coverslipped with ProLong antifade reagent with DAPI (Molecular Probes). Whole sections were imaged with an Olympus VS120 slide scanning microscope. Tyrosine hydroxylase (TH) and Dopamine transporter (DAT) were stained using standard staining protocol (primary antibodies: Mouse monoclonal anti-TH (Immunostar cat. no. 22941; RRID:AB_572268), Rat monoclonal anti-DAT (Millipore cat. no. MAB369; RRID: AB_2190413); secondary antibodies: Goat anti-mouse IgG Alexa Fluor 647 and Goat anti-rat IgG Alexa Fluor 647 (Thermo Fisher Scientific cat. no. A21236; RRID: AB_2535805; and cat. no. A21247; RRID: AB_141778)). To quantify fluorescence intensity in Extended Data Fig. 1e, three coronal sections were chosen for each mouse at AP levels +1.5, +1.0 and +0.5 mm from bregma. Regions of interest of the same size were drawn in the left cortex and the left (intact) and right (lesioned) dorsal striatum. The mean fluorescence intensity of DAT in striatum was measured and normalized to that of cortex for comparison. To estimate the penetrance of stGtACR2 in SNc DA neurons (Fig. 3b), we used Cellpose^49^ to automatically segment the cell bodies of TH-positive neurons in epifluorescence images of ventral midbrain, excluding VTA. We calculated the mean fluorescence intensity of stGtACR2 in DA neurons in ImageJ^50^ using masks obtained with CellPose. The fraction of TH-positive cells also positive for stGtACR2 was estimated by calculating the fraction of TH-positive cell masks where stGtACR2 fluorescence intensity exceeded a background threshold common to all sections.

### Statistics and reproducibility

Statistical analyses were performed using Prism 10 (GraphPad), unless specified otherwise. All statistical tests were two-sided. Normally distributed data (assessed using Shapiro–Wilk test) were compared with the following parametric statistical tests (as indicated in the text): one-sample *t*-test for comparison with a hypothetical mean of 0% change, two-sample *t*-test (with Welch’s correction) for comparisons between unpaired data points, paired *t*-test for comparisons between paired data points, repeated-measure one-way analysis of variance (ANOVA) with Geisser–Greenhouse correction followed by Dunnett’s multiple comparison tests for comparisons between multiple groups with balanced data and mixed-effect model with post hoc Dunnett’s tests for comparisons between multiple groups with unbalanced repeats. In Fig. 3, when multiple sessions of the same type were performed in the same mice, we used linear mixed-effect models with the fitlme function in MATLAB: *y* ≈ 1 + manipulation + (1 | mouse ID) + (1 | mouse ID:session number). This tests for the significance of an experimental manipulation (that is, optogenetic versus sham light) as fixed effect, with mouse number and recording session per mouse assigned as random effects. The following nonparametric tests were used for non-normally distributed data: Wilcoxon signed rank-sum test for comparisons between paired data points and Mann–Whitney test for comparisons between unpaired data points. *N* represents the number of mice and *n* the number of sessions, lever presses or imaged neurons, unless indicated otherwise. Paired data are linked by gray lines. Summary population data across groups are reported in text and figures as mean ± s.e.m., with shaded areas and error bars in figures representing s.e.m. Exact *P* values are provided in text and figure legends, and statistical significance in figures is presented as *P < 0.05, **P < 0.01, ***P < 0.001 and ****P < 0.0001. No statistical method was used to predetermine sample size. The investigators were not blinded to allocation during experiments and outcome assessment.

### Reporting summary

Further information on research design is available in the Nature Portfolio Reporting Summary linked to this article.

## Online content

Any methods, additional references, Nature Portfolio reporting summaries, source data, extended data, supplementary information, acknowledgements, peer review information; details of author contributions and competing interests; and statements of data and code availability are available at 10.1038/s41593-025-02102-1.

## Supplementary information

Reporting Summary

## Source data

Source Data Fig. 1 Source data for graphs shown in Fig. 1.

Source Data Fig. 2 Source data for graphs shown in Fig. 2.

Source Data Fig. 3 Source data for graphs shown in Fig. 3.

Source Data Extended Data Fig. 1 Source data for graphs shown in Extended Data Fig. 1.

Source Data Extended Data Fig. 2 Source data for graphs shown in Extended Data Fig. 2.

Source Data Extended Data Fig. 3 Source data for graphs shown in Extended Data Fig. 3.

Source Data Extended Data Fig. 4 Source data for graphs shown in Extended Data Fig. 4.

## Data Availability

The data that support the findings of this study are available from the corresponding author on reasonable request. Source data are provided with this paper.

## Code availability

All code related to this study was developed in MATLAB and is available at https://github.com/TritschLab/Liu-etal-2025.
